# Prognosis of hyperviscosity syndrome in newly diagnosed multiple myeloma in modern-era therapy: A real-life study

**DOI:** 10.3389/fimmu.2022.1069360

**Published:** 2022-12-08

**Authors:** Pierre-Edouard Debureaux, Stéphanie Harel, Nathalie Parquet, Virginie Lemiale, Virginie Siguret, Laurie Goubeau, Florence Morin, Bruno Royer, Wendy Cuccuini, Dikelele Elessa, Floriane Theves, Anne C. Brignier, Elie Azoulay, Bertrand Arnulf, Alexis Talbot

**Affiliations:** ^1^ Immuno-Hematology, Saint Louis Hospital, Paris, France; ^2^ Apheresis Center, Saint Louis Hospital, Paris, France; ^3^ Medical Intensive Care Unit (ICU) unit, Saint Louis Hospital, Paris, France; ^4^ Hematology Laboratory, Lariboisière Hospital, Paris, France; ^5^ Immunology Laboratory, Saint Louis Hospital, Paris, France; ^6^ Cytogenetic laboratory, Saint Louis Hospital, Paris, France; ^7^ Medical School, Université Paris Cité, Paris, France

**Keywords:** HVS, myeloma, prognosis, TPE, neurological

## Abstract

Hyperviscosity syndrome (HVS) is a rare complication of newly diagnosed multiple myeloma (NDMM) related to high tumour burden. Studies about the prognosis of HVS in modern-era therapy for NDMM are missing. We investigated a retrospective cohort study of NDMM with HVS between 2011-2021. Thirty-nine NDMM patients with HVS were included. HVS presentation was heterogeneous, with asymptomatic, mild, and neurological forms in 23%, 59%, and 18% of cases, respectively. No thrombosis or major bleeding was observed. Therapeutic plasma exchanges were used in 92% of patients, which were effective and well tolerated. No rebound effect was observed. All patients except one had at least one CRAB criterion. Most of the patients received bortezomib and high-dose steroids (95%) associated with an immunomodulatory drug (43%) or alkylating agents (42%). HVS in NDMM patients had dismal overall survival matched to multiple myeloma patient controls (without HVS) in our center (median: 3.6 vs. 7.7 years, p=0.01), as confirmed by multivariate analysis. Early deaths (in the first two months) occurred in 21% of older patients (>65 years). HVS in NDMM patients is a rare but life-threatening complication associated with high lethality in older patients and be a potential dismal prognosis factor in the modern treatment era.

## Introduction

Multiple myeloma (MM) is a plasma cell–derived neoplasm characterized primarily by the abnormal production of monoclonal immunoglobulin (M-protein) found in serum or urine and resulting in various complications (1). The first prognosis scores and treatment criteria were based on tumour mass and related complications, with the Salmon & Durie classification published in 1975 (2). Later, the treatment criteria evolved to be based on principal complications of high tumour burden as summarized in the CRAB acronym: hyperCalcemia, Renal insufficiency, Anemia, and Bone lesions and the SLiM acronym: greater than or equal to Sixty percent clonal plasma cells in the bone marrow, involved/uninvolved free Light chain ratio of 100 or more with the involved FLC being greater than or equal 100 mg/L and MRIM with more than one focal marrow lesion (3). More rarely, other manifestations of high tumour burden such as hyperviscosity syndrome (HVS) can be present at MM diagnosis.

HVS results from a high blood viscosity due to an abnormal protein or cellular element increase, frequently secondary to plasma cell disorders with immunoglobulin (Ig) (4). The clinical triad of HVS includes mucosal bleeding (epistaxis, gum), visual abnormalities (blurring, papilledema, retinal bleeding, venule thrombosis), and neurological abnormalities (drowsiness, ataxia, cerebral bleeding) (4). In a context of HVS suspicion or for asymptomatic patients with a high protein concentration, the diagnosis can be confirmed by ophthalmologic examination. On one hand, the principal cause of HVS is pentameric IgM in the context of Waldenström macroglobulinemia (WM). At diagnosis, 40% of WM patients present with HVS, which is a criterion for starting specific treatment (5, 6). On the other hand, MM plasma cells produce mostly monomeric IgG or dimeric IgA M-protein, leading to fewer blood viscosity increased than pentameric IgM. Among MM patients, 3–6% experienced this complication at diagnosis in cohorts prior to the 21^st^ century (7, 8).

To avoid vital complications (thrombosis, gut, or cerebral bleeding), early management with therapeutic plasma exchange (TPE) for HVS is advised as well as management in the intensive care unit (ICU) for severe forms (neurological signs, bleeding, or thrombosis related to HVS) (9, 10). One TPE can be sufficient to resolve symptoms of HVS (10). IgG is presented mainly in the intravascular compartment (45–65%), but the turnover rate from extravascular to intravascular is 2% per hour, which could require multiple TPE until HVS symptoms resolve (5). In the absence of concurrent effective chemotherapy for MM, a rebound phenomenon was described as related to persistent M-protein production (10). For long-term management, therapy to control the underlying disease is required. Before 2014, HVS was a criterion for initiating treatment (3). In 2014, the international consensus of the International Myeloma Working Group (IMWG) for MM withdrew HVS as a treatment criterion based on the low occurrence of HVS (8) and the redundancy between indicators of HVS and CRAB (1).

The largest cohort of MM with HVS (including 10 IgG MM patients and a review of 51 patients from the literature) was published in 1972 and reported an HVS incidence of 4% for IgG MM (7). In a more recent monocentric cohort of 170 consecutive patients with MM from 2009–2010, HVS was present in five patients at diagnosis (incidence of 3%) (11). In parallel, the prognosis of newly diagnosed MM (NDMM) patients has dramatically improved by the arrival of new drugs tested in recent clinical trials (12, 13). Also, recent clinical trials had not included NDMM with HVS related to an aggressive presentation, because inclusion in these trials often required a washout of 15 days after the last TPE. With the current treatment revolution in MM, there are no recent data about the impact of HVS on MM prognosis. Our objective is to describe the outcomes of NDMM with HVS in a recent and real-life setting and to evaluate the prognosis impact of this initial complication.

## Methods

### Patients and definition

A retrospective monocentric study was performed at Saint Louis Hospital, Assistance Publique – Hôpitaux de Paris (AP-HP), Paris, France between 2011–2021 to include NDMM patients with HVS. Inclusion criteria were patients aged ≥18 years with NDMM and HVS confirmation based on abnormalities related to HVS at ophthalmologic examination (performed for clinic symptoms of HVS or at the discretion of the physician for patients who had high M-protein) or severe clinical forms (neurological symptoms, bleeding, or thrombosis related to HVS). Patients were excluded when HVS did not occur at MM diagnosis. The control patients were NDMM patients without HVS and were selected from multidisciplinary meeting records of the same period matched on sex, age ( ± 5 years), year of MM diagnosis ( ± 3 years), and first-line regimen of MM treatment with a ratio of one HVS for two controls. The estimate of the incidence of HVS was calculated from the EMMY registry, listing the lines of treatment initiated for MM every year over a period of three months since 2017 in our center.

Multiple myeloma definitions (diagnosis, treatment criteria, response, relapse) were based on the recommendations of the International Myeloma Working Group (IMWG) (1, 14). Primary plasma cell leukaemia (pPCL) was defined by at least 20% circulating plasma cells and a total plasma cell count in peripheral blood of at least 2×10^9^/L. High-risk cytogenetics was described as the presence of translocations t (4, 14) or del(17p) by fluorescence *in situ* hybridization (FISH) according to the consensus from the period of 2011–2021 (15, 16).

PT (prothrombin time) with no dilution, factor II, V, VII, X (dilution of 1/10) were measured using Neoplastin CI+ (Stago) on a STAR Analyser. Fibrinogen was measured using STA-liquid fibrinogen. We chose to express PT results as an international normalized ratio (INR), as it leads to better standardization of results across the various reagent batches used during the study period.

The AP-HP Data Protection Office approved our protocol and registered our database. Patient data were obtained in conformity with the Declaration of Helsinki.

### Objectives and statistical analysis

The duration of follow-up was calculated using the inverse method (17). Patients were excluded from survival analysis if they had less than three months of follow-up. Overall survival (OS) was calculated from the MM diagnosis until death or the last follow-up (whichever occurred first). Time to next treatment was calculated as the time from the start of front-line therapy to an event (start of a new line of treatment, death, or last follow-up; whichever occurred first). The primary outcome was OS. Categorical variables were compared using a χ^2^ test or Fisher’s exact test, as appropriate. Continuous variables were compared using a Student’s t test or a Wilcoxon rank-sum test. Kaplan–Meier curves were plotted for survival, and data for various groups were compared using a log-rank test. To assess factors independently associated with overall survival, a Cox model was performed with variables which reach p < 0.10 in univariate analysis. All statistical tests were two-tailed with a significance level of 0.05. Statistical tests were performed using R software version 4.0.4 (https://www.r-project.org) and Prism version 9.2.0.

## Results

### NDMM with HVS cohort

**Figure 1:**
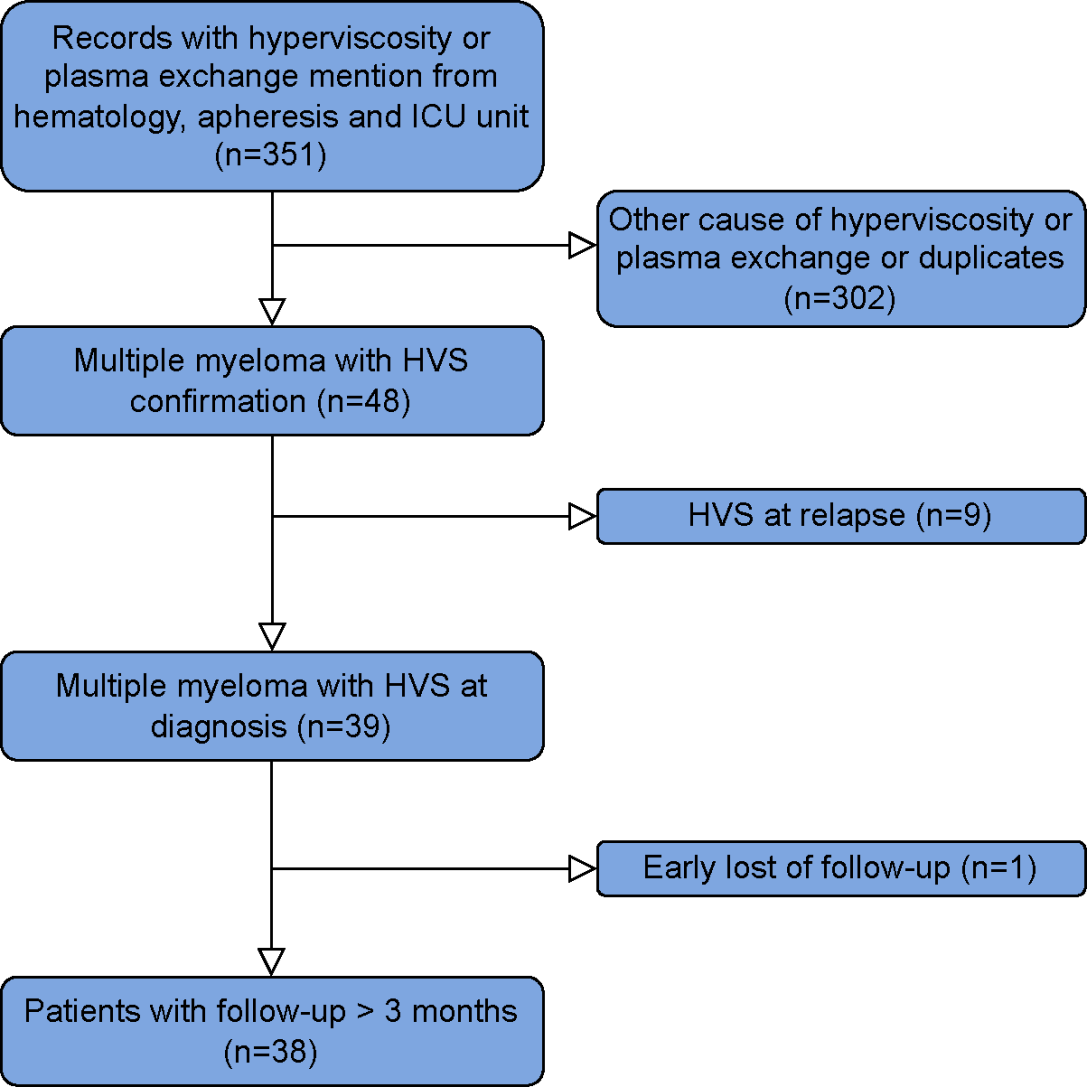
Flowchart of the study.

Among the 46 cases of MM with suspected HVS referred at Saint Louis Hospital, Paris, France, between 2011–2021, 39 (85%) patients with HVS confirmation were included (**Figure 1**). Seven patients had HVS occurrence in subsequent lines of therapy and were excluded. The HVS incidence in NDMM was 3.4% based on the estimated rate of 110 NDMM diagnosed per year in our center (EMMY registry).

The median age for MM patients with HVS at diagnosis was 59 years (IQR 51–68, **Table 1**). The main isotype of Ig was IgG (69%, including seven IgG1 and one IgG4 among the eight patients with available subclass data), followed by IgA (28%). The only patient without CRAB criteria had only one SLiM criterion with two focal lesions based on magnetic resonance imaging. The most frequent cytogenetic abnormally was t (11, 14), which was observed for 37% of the patients.

**Table 1:** Initial characteristics of NDMM with HVS.

	Total N=39
Men	26 (67%)
Age at diagnosis (years)	59 [51–68]
> 65 years	14 (36%)
MGUS history	9 (23%)
**Type of immunoglobulin (Ig)**	
IgG	27 (69%)
IgA	11 (28%)
IgM	1 (3%)
Light chain type: Kappa/Lambda	24 (62%)/15 (38%)
Symptomatic plasma cell disorder	39 (100%)
Plasma cell leukaemia	2 (5%)
Plasma cells in bone marrow (%)	34 [14–68]
Haemoglobin level (g/L)	78 [67–85]
Platelets (10^9^/L)	147 [106–199]
Calcemia (mmol/L)	2.3 [2.2–2.6]
Creatinine level (µmol/L)	113 [85–207]
Proteinuria (normalized by creatinuria level, g/mmol)	150 [50–393]
**CRAB criteria**	38 on 39 (97%)
Hypercalcemia	11 (28%)
Renal injury	13 (33%)
Anemia	36 (92%)
Lytic Bone lesion	17 (44%)
**SLiM criteria**	
Light chain ratio > 100*	14 of 19 (74%)
More than one focal lesion on MRI > 10 mm	15 of 30 (50%)
Plasma cell bone marrow infiltration ≥ 60%	12 (32%)
Extramedullary localization	2 (5%)
**Cytogenetics**	38 of 39
**High risk cytogenetic**	11 of 38 (29%)
t(4;14)	7 of 38 (18%)
Del (17p)	4 of 38 (11%)
t(11;14)	14 of 38 (37%)
q+	7
Del (1p32)	3
t(14;16)	2
**ISS score**	
1-Low	2 of 33 (6%)
2-Intermediate	13 of 33 (39%)
3-High	18 of 33 (55%)
**R-ISS score**	
1-Low	1 of 33 (6%)
2-Intermediate	24 of 33 (70%)
3-High	8 of 33 (24%)

Continuous variables are presented with %. Numerical variables are presented with median and IQR. MGUS: monoclonal gammopathy of undetermined significance, * involved/uninvolved free light chain ratio of 100 or more with the involved FLC greater than or equal 100 mg/L, MRI, Magnetic resonance imaging; ISS, international system score; R-ISS, revised ISS.

### HVS diagnosis and management

**Figure 2:**
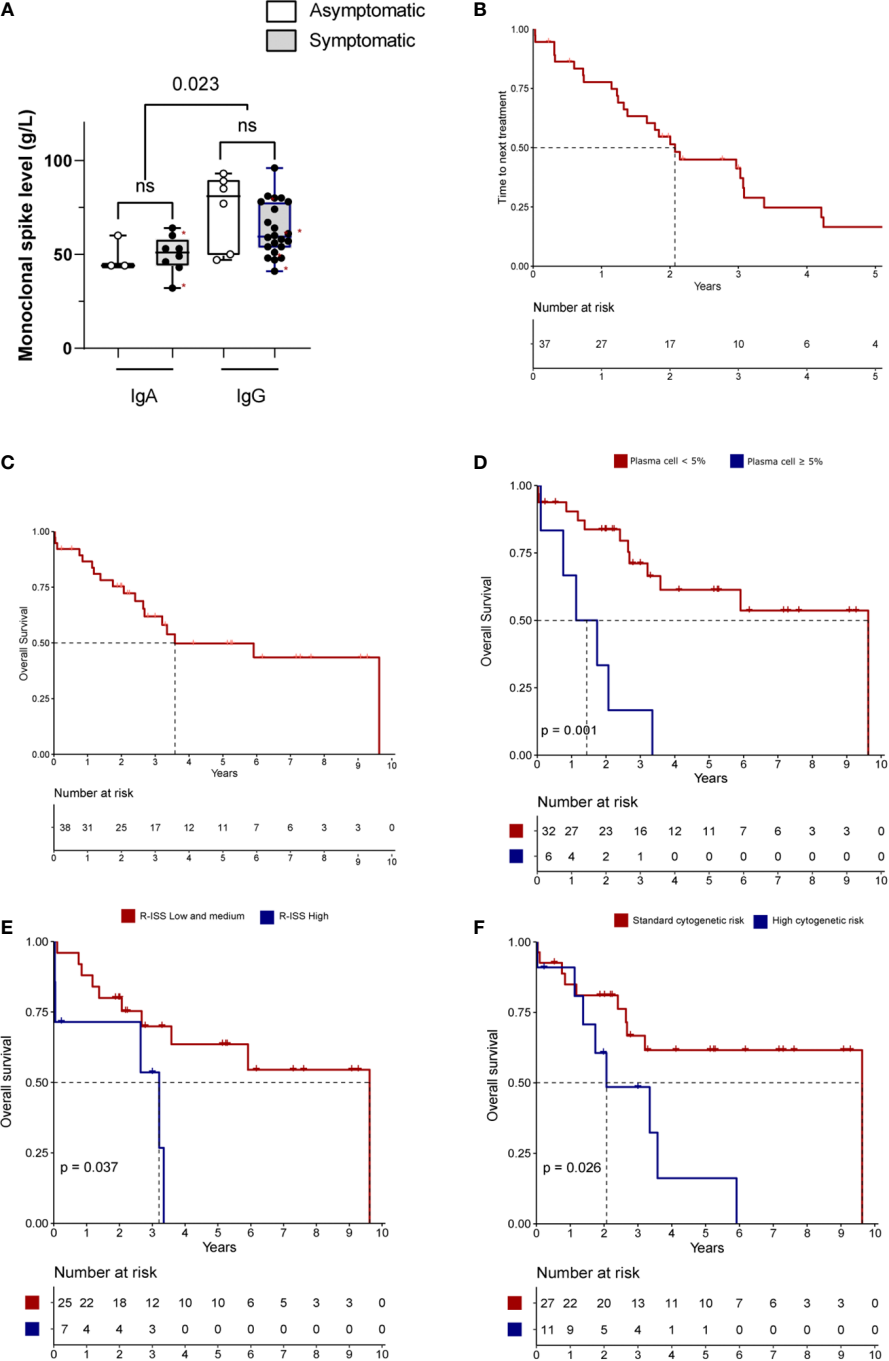
Description and outcomes of HVS NDMM **(A)** Monoclonal protein level between Ig type and HVS symptoms for NDMM. Red stars denote severe cases with neurological symptoms. **(B–F)** Survival of HVS NDMM patients. Time to next treatment **(B)** and overall survival **(C)** of NDMM with HVS. **(D–F)** Overall survival based on presence of plasma cells in blood by cytology analysis **(D)**, score R-ISS (low=R-ISS1 and R-ISS2; high=R-ISS3) **(E)** and cytogenetic risk (high risk= presence of t(4;14) or del(17p) by FISH; standard risk=others) **(F)**. Number of patients could vary related to missing data. NS, not significant.

Concerning clinical form, the presentation of HVS was heterogenous (**Figure 2A**; **Table 2**): 23% were clinical asymptomatic, 59% had mild clinical signs (43% epistaxis, 33% blurry vision, and 23% headache) and 18% had a severe form with neurological symptoms. No major ischemic or bleeding was observed at diagnosis. Fifteen (38%) patients required ICU admission: a severe form of HVS for six, other complications associated with NDMM (two acute kidney injury, three sepsis or/and two acute respiratory distress syndromes) for six, and only for TPE management for three.

**Table 2:** Description of HVS for NDMM.

	No TPE *N=3*	TPE *N=36*	Total *N=39*
Hospitalization-HVS diagnosis delay (days)	0 day [0–0]	1 day [0–2]	1 day [0–2]
**Clinical symptoms of HVS**	3 (100%)	27 (75%)	30 (77%)
Epistaxis	2 (67%)	15 (42%)	17 (44%)
Blurry vision	2 (67%)	11 (31%)	13 (33%)
Headache	2 (67%)	7 (19%)	9 (23%)
Tinnitus	1 (33%)	7 (19%)	8 (21%)
Alteration of mental state	0	7 (19%)	7 (18%)
Vertigo	1 (33%)	1 (3%)	2 (5%)
**Ophthalmologic diagnosis of HVS**	3 (100%)	29 (81%)*	32 (82%)
Delay veinula clearance	0	21 (58%)	21 (54%)
Retinal hemorrhage	2 (67%)	8 (22%)	10 (26%)
Papillary edema	0	4 (11%)	4 (10%)
Retinal vein occlusion	1 (33%)	1 (3%)	2 (5%)
**Hemostasis disorder related to HVS**			
PT expressed in INR	1.3 [1.1–1.3]	1.4 [1.2–1.6]	1.4 [1.2–1.6]
Fibrinogen (g/L)	3.6 [1.7–4]	2.6 [2.1–3.9]	2.6 [2.1–3.9]
HVS diagnosis to TPE initiation delay (days)	ND	1 [0–1]	1 [0 – 1]
**Number of TPE sessions**	ND	2 [1–2]	2 [1 – 2]
1	ND	15 (42%)	15 (38%)
2	ND	19 (53%)	19 (48%)
3	ND	2 (5%)	2 (5%)
Hospitalization duration (days)	9 [9–14]	13 [10–23]	13 [10–21]

Continuous variables are presented with %. Numerical variables are presented with median and IQR.

TPE, therapeutic plasma exchange; HVS, hyperviscosity syndrome; PT, prothrombin time; INR, international normalized ratio; ND, no data available.

*Five examinations were missing related to emergency (neurologic or hemostasis complication), one unknown cause and one negative (presence of diabetic retinopathy, which impaired ophthalmic conclusion).

Thirty two of 33 patients (96%) with ophthalmic examination had confirmed HVS diagnosis with mainly delayed venule clearance (56%), followed by retinal haemorrhage (26%). Justification of ophthalmic examination omission was emergency treatment (n=6, 16%) based on neurological complications for two, high INR for two, and unknown reason for one.

Higher levels of monomeric IgG were observed in NDMM patient with HVS compared with dimeric IgA (median 61 vs. 49 g/L, p=0.023, **Figure 2A**). No difference in Ig levels was observed between asymptomatic and symptomatic forms (IgG p=0.31; IgA p=0.80). Among patients with IgG NDMM (n=27), the presence of t (11, 14) translocation was associated with a higher monoclonal spike (median: 71 vs. 59 g/L, p=0.0001). Moderate coagulation abnormalities were observed in NDMM with HVS, with slightly elevated INR (median 1.4), along with slightly decreased levels of factor II, V, VII and X. Fibrinogen levels in NDMM with HVS was normal in median (2.2 g/L), but eight patients (21%) had fibrinogen below 2 g/L. Furthermore, median fibrinogen levels for IgA NDMM were lower than IgG NDMM (2.2 vs. 3.3 g/L, p=0.011) without difference for INR.

The treatment of HVS was based mainly on TPE for 36 patients (92%), but three patients only received steroids. Patients who received TPE have a concomitant administration of high dose steroid. After treatment, these three patients with mild HVS slightly improved clinical signs and protein levels (median -10% at ten days, **Supplementary Figure 1A**). One, two, or three sessions of TPE were performed in 15 (42%), 19 (53%), and 2 (5%) patients, respectively. Twenty-one patients (58%) received TPE in the apheresis center with centrifugation, 12 (33%) in the ICU with filtration, and 3 (9%) in both units. Fluid substitution was albumin 5% alone for 60%, and albumin 5% + fresh frozen plasma (FFP) for 40% of the patients. The session was well tolerated for 58 of 59 TPE sessions. One patient experienced a transitory hypotension during a session that was quickly resolved after fluid resuscitation. Multimodal therapy (TPE + steroid + anti-plasma cell treatment) efficacy was confirmed for all patients from both clinical (resolution of symptoms in hours after TPE) and biological perspectives (**Supplementary Figure 1B**).

One month after HVS diagnosis, clinical and biological features related to HVS were improved in 33 patients (84%). Six patients (16%) had sequela or complications related to HVS: three (8%) early deaths and three (8%) ophthalmologic sequelae. Three thromboses occurred for three patients during the front-line treatment by bortezomib + cyclophosphamide + dexamethasone (VCD): one acute myocardial ischemia and two deep venous thrombosis.

No rebound effect was observed at one month post TPE with a median protein level decrease of 66% (IQR 60–73, **Supplementary Figure 1C**). Only one patient had increased protein level after one month of therapy related to a refractory status at the front-line treatment without HVS recurrence.

### Multiple myeloma treatment and outcomes

Most of the patients received triplet association with a backbone of bortezomib and high dose steroids (95%) associated with an immunomodulatory drug like thalidomide (21%), lenalidomide (34%), or alkylating agents like cyclophosphamide (37%) or melphalan (5%) (**Supplementary Table 1**). The anti-plasma cell treatment was started after the end of TPE sessions. If TPE washout criteria was not considered, two-thirds of these patients met the eligibility criteria for the most recent clinical trials for young (MIDAS study, n=16 [NCT04934475]) and older patients (BENEFIT study, n=9 [NCT04751877]). Nevertheless, only two patients (8%) were included in a clinical trial. One patient did not receive MM treatment except TPE and steroids related to multiple septic shocks and died one month after the HVS diagnosis.

The overall response rate after front-line treatment, defined as partial response (PR) or better, was 92% (n=35). The median time to the next treatment was 2.1 years (95%IC 1.4–3.4, **Figure 2B**). During the following therapy sequence, six patients had HVS recurrence at MM relapse.

With a median follow-up of 5.2 years, the median OS in our cohort was 3.6 years (95%CI 2.7–not reached, **Figure 2C**). Eighteen patients (47%) died during the follow-up, including three early deaths (one septic shock, one pneumonia and one liver failure) two months after the diagnosis in older patients (>65 years), thirteen deaths with active MM and two deaths related to transplant-related toxicity (one post-auto-hematopoietic stem cell transplantation [HSCT] and one post-allo-HSCT) (**Supplementary Table 2**). Among the 14 patients over 65 years old, the early mortality rate (<3 months) was 21%.

In univariate analysis (**Figures 2D-F**; **Supplementary Table 3**), the presence of >5% of blood plasma cells evaluated by cytology, R-ISS3 score, high-risk cytogenetics, LDH above normal level shortened the OS. The multivariate analysis could not be performed related to a low number of events.

### Control cohort of NDMM without HVS

**Figure 3:**
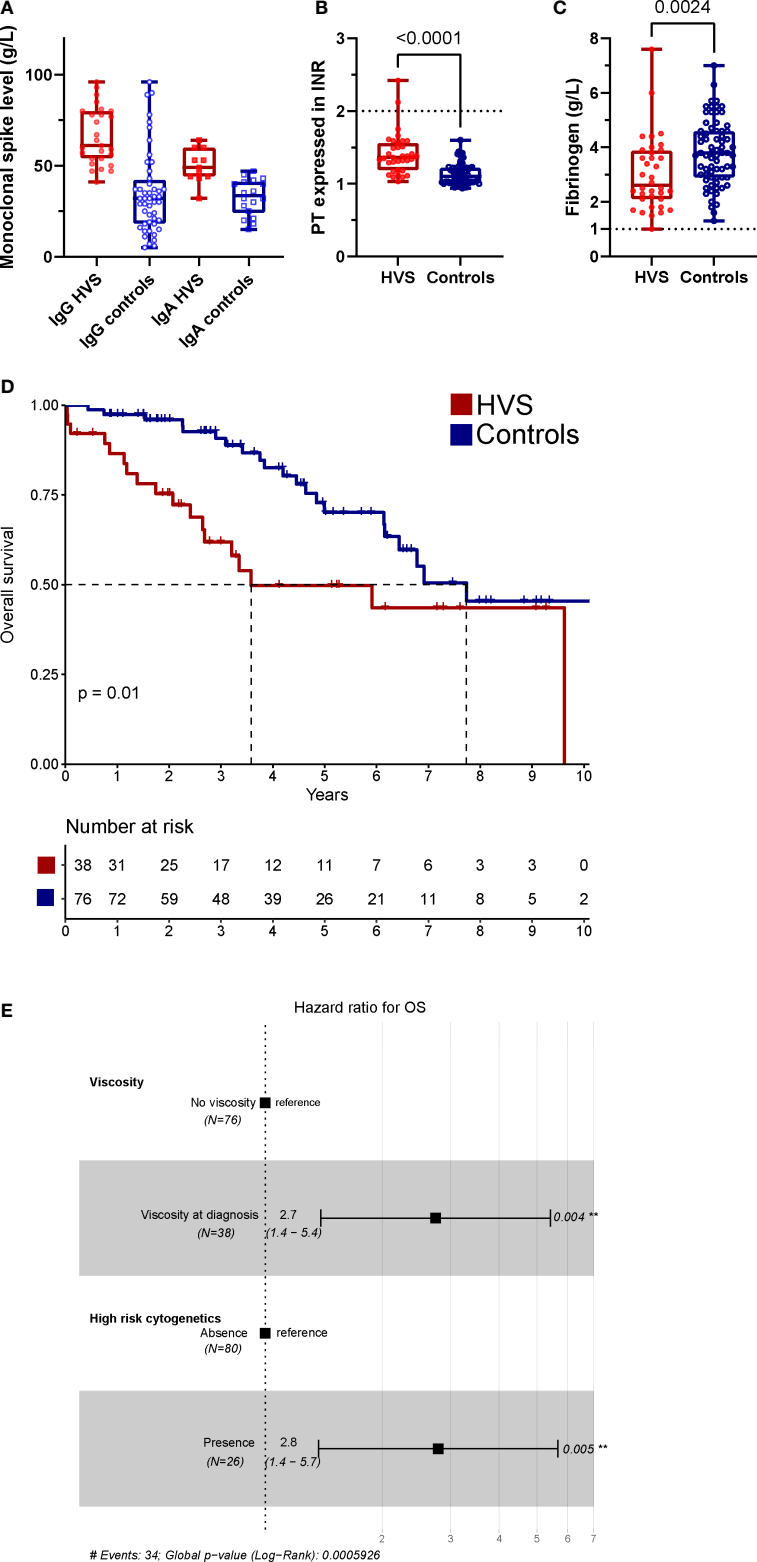
Comparison to NDMM controls. **(A)** Monoclonal protein level between HVS and controls for each sub-type **(B, C)** PT expressed in INR **(B)** and fibrinogen **(C)** levels between HVS and control NDMM. Line represents in each figure the cut-off of increase bleeding risk in clinical **(D)** OS of HVS (red) and controls (blue) patients, **(E)** Forest plot for OS with final model. In univariate, HVS, cytogenetic risk, ISS and R-ISS have a p < 0.1. Each patient with NDMM and HVS was paired with two NDMM controls without HVS based on sex, age ( ± 5 years), year of diagnosis ( ± 3 years), and first-line MM therapy. The median follow-up for the controls (n=76) was five years (IQR 2.7–8).

In comparison with NDMM control (n =76, median follow-up of 5 years, IQR 2.7-8), no difference was observed for paired criteria, cytogenetics, and percentage of auto-HSCT performance (**Table 3**; **Supplementary Table 2**). NDMM patients with HVS had higher M-protein level at diagnosis (**Figure 3A**), higher β2-microglobulin level, lower albumin level, higher plasma cell infiltration in bone marrow, higher ISS3 rate, higher INR and lower fibrinogen compared to NDMM controls (**Figures 3B, C**). M-protein levels correlated with INR (cor -0.57, p<0.001) and fibrinogen level (cor -0.35, p<0.001). HVS in NDMM patients had dismal OS compared to controls (median 3.6 vs. 7.7 years, p=0.01, **Figure 3D**). In a multivariate analysis of pooled HVS and controls, HVS (HR 2.7; 95%CI 1.4–5.4, p=0.004) and high-risk cytogenetics (HR 2.8; 95%CI 1.4–5.7, p=0.005) were the only two independent adverse factors for OS (**Figure 3E**). HVS remained an independent factor for OS for NDMM with the addition of the ISS score or R-ISS score.

**Table 3:** Comparison between NDMM with or without HVS (ratio 1:2).

	HVS NDMM *N=38*	Control *N=76*	p-value
Men	26 (68%)	47 (62%)	0.63
Age at diagnosis	60 [50–68]	61 [54–69]	0.48
Year of diagnosis	2017 [2014–2018]	2015 [2013–2018]	0.12
**Type of Immunoglobulin (Ig)**			0.11
IgG	26 (68%)	48 (63%)	
IgA	11 (29%)	18 (24%)	
IgM	1 (3%)	0	
Light chain	0	10 (13%)	
Monoclonal protein (g/L)	59 [49–76]	30 [15–39]	**<0.001**
β2-microglobulin (mg/L)	6 [4–11]	4 [3–5]	**0.001**
Albumin (g/L)	30 [25–34]	37 [32–42]	**<0.001**
LDH above upper limit	6 (16%)	14 (18%)	0.72
Bone marrow plasma cell (%)	35 [14–69]	17 [12–32]	**0.013**
**Hemostasis**			
PT expressed in INR*	1.4 [1.2–1.6]	1.1 [1–1.2]	**<0.001**
Fibrinogen (g/L)	2.6 [2.1–3.9]	3.8 [2.9–4.6]	0.002
**Cytogenetics**			
**High-risk cytogenetics**	11 (29%)	15 (20%)	0.41
t(4;14)	7 (18%)	11 (15%)	0.81
Del (17p)	4 (11%)	4 (5%)	0.44
t(11;14)	14 (37%)	18 (24%)	0.23
**ISS score**	32 of 38 (84%)	59 of 76 (78%)	0.41
1-Low	2 (6%)	19 (32%)	**0.003**
2-Intermediate	13 (41%)	26 (44%)	
3- High	17 (53%)	14 (23%)	
**R-ISS score**	32 of 38 (84%)	58 of 76 (77%)	0.33
1- Low	1 (3%)	12 (21%)	0.06
2-Intermediate	24 (75%)	38 (66%)	
3-High	7 (22%)	8 (14%)	
**Front line therapy**			0.40
(V)RD	11 VRD (29%)	18 VRD and 5 RD (30%)	
VTD	7 (18%)	18 (24%)	
VCD	14 (37%)	23 (30%)	
VMP	2 (5%)	4 (5%)	
DaraVRD	2 (5%)	4 (5%)	
PAD – VCD	1 (3%)	2 (3%)	
Other	1 (3%)	2 (3%)	
AutoHSCT realization	22 (58%)	39 (51%)	0.64

Continuous variables are presented with %. Numerical variables are presented with median and IQR. AutoHSCT, autologous hematopoietic stem cell transplantation; ISS, international score system; PT, prothrombin time; INR, international normalized ratio; *normal INR, < 1.25; R-ISS, revised ISS; VCD, bortezomib + cyclophosphamide + dexamethasone; VRD, bortezomib + lenalidomide + dexamethasone; VTD, bortezomib + thalidomide + dexamethasone; VMP, bortezomib + melphalan + dexamethasone; PAD, bortezomib + adriamycin + dexamethasone. Bold text is for headline in left column and positive test (p < 0.05) for the right one.

## Discussion

To our knowledge, we report here the most extensive study of NDMM patients with HVS in the modern era therapy. HVS was rare, with an incidence of 3.4% at MM diagnosis, but a life-threatening complication in the short term in older patients and with a long-term impact on all patients.

Most cohorts (including ≥5 patients) of MM with HVS were published in the 1970s when no efficient treatment was available for MM, which explained the higher occurrence of symptomatic HVS (5–6%) (7, 18). After 1980, only a Mexican series of five patients was presented (19). Some cohorts (18, 20) observed that IgG3 subclass was associated with HVS in IgG myeloma, which was not confirmed (7, 21). We did not find any NDMM with IgG3 subclass associated with HVS based on eight IgG myeloma tested (29% of the IgG MM). For clinical presentation, our cohort was similar to a previous report (majority of mild signs and 10–20% of severe forms with neurological signs) and similar to recent report of HVS in 997 WM (22). Haemostasis disorder with HVS in NDMM was confirmed in our case-controls analysis related to lower fibrinogen level and slightly elevated INR, both likely related to haemodilution (correlation with M-protein level). Compared with the historical cohort of MM with HVS (7, 18), no severe bleeding or thrombosis was presented related to mild haemostasis disorder observed in our NDMM with HVS cohort. Moreover, this improvement could be explained by a better recognition and management of HVS based on TPE and absence of rebound effect related to more efficient MM treatment. TPE international recommendation for HVS was exclusively based on WM experience (10). We saw the efficacy of TPE associated with anti-plasma cell therapy with 86% of full recovery after one or two sessions and a good safety profile (no severe reaction during procedure). In the IMWG recommendation of 2014 (1), HVS was withdrawn because of low incidence and a systematically association with CRAB criteria. This recommendation was not based on recent publications, but our cohort confirmed these two assumptions.

Nevertheless, HVS in the modern therapeutic era for NDMM (median OS at 3.6 years) was associated with dismal prognosis compared to the control group from the same institution (median OS at 7.7 years, similar to other real life cohort (23)) with adjustment of cytogenetics and prognosis score. This is a major finding which was not confirmed for HVS in WM (OS of 12 vs. 12 years with or without HVS, p=0.63) (22). This study found that bone marrow plasma cell infiltration, serum M-protein level, β2-microglobulin, and albumin were different between HVS and control NDMM patients. Most of these variables are direct or indirect measures of the tumour burden, the basis of the historic prognosis classification of Durie & Salmon (2). Compared with more recent prognosis score, NDMM with HVS in our cohort had a similar OS at five years than R-ISS3 (~45%) (16). Recent TPE is a frequent exclusion criterion in MM clinical trials and explains our cohort’s low inclusion rate. Related to the unfavourable prognosis of HVS, including these patients in clinical trials seems necessary. Furthermore, the use of antibodies against CD38 has completely modified the standard of care in the front-line of MM and need to be tested in HVS NDMM (only two patients in our cohort received it) (12, 13).

This study is limited by the monocentric retrospective design. It can be challenging to conduct adequate prospective studies in a rare complication of NDMM and frequently excluded from clinical trial that why a real-life monocentric study can address primary interrogation but a multicentric prospective cohort could confirm it. A single-center study can reduce the bias of treatment heterogeneity over ten years for the case-control analysis. Others potential limitations were the absence of serum viscosity measurement, despite the absence of proven correlation between HVS symptoms and gravity (4, 22), and extensive haemostasis analysis. Despite confirmation of specific haemostasis disorder, no severe complication was observed, which can be explained by a quick recognition and management of HVS in a tertiary center. Based on our results, further studies should specifically address haemostasis disorders in the context of HVS like disparity between IgG and IgA NDMM with HVS. Finally, controls were not matched for cytogenetic and prognosis scores. We mitigate this effect with the multivariate analysis which confirmed the dismal prognosis of HVS.

Our study demonstrates that HVS for NDMM patients is a rare but life-threatening complication associated with early death in older patients. HVS seems associated with dismal survival in multiple myeloma but additional study and cohort are needed to confirm the results. NDMM patients with HVS need to be included in clinical trials and biological bank to confirm our results.

## Data availability statement

The original contributions presented in the study are included in the article/**Supplementary Material**. Further inquiries can be directed to the corresponding author.

## Ethics statement

Ethical review and approval was not required for the study on human participants in accordance with the local legislation and institutional requirements. Written informed consent for participation was not required for this study in accordance with the national legislation and the institutional requirements.

## Author contributions

Conception and design: P-ED, SH, AT. Collection and assembly of data: P-ED, SH, NP, VS, LG, FM, WC, DE, FT, AT. Data analysis and interpretation: P-ED, SH, BR, VS, BA, AT. Taking care of patients: P-ED, SH, NP, VL, BR, DE, FT, AB, EA, BA, AT. Article writing: P-ED, SH, BR, AT. All authors read and approved the final version of this manuscript.

## References

[1] Rajkumar SV Dimopoulos MA Palumbo A Blade J Merlini G Mateos M-V. International myeloma working group updated criteria for the diagnosis of multiple myeloma. Lancet Oncol (2014) 15:e538–48. doi: 10.1016/S1470-2045(14)70442-5 25439696

[2] Durie BG Salmon SE. A clinical staging system for multiple myeloma. correlation of measured myeloma cell mass with presenting clinical features, response to treatment, and survival. Cancer (1975) 36:842–54. doi: 10.1002/1097-0142(197509)36:3<842::aid-cncr2820360303>3.0.co;2-u 1182674

[3] Group TIMW. Criteria for the classification of monoclonal gammopathies, multiple myeloma and related disorders: a report of the international myeloma working group. Br J Haematol (2003) 121:749–57. doi: 10.1046/j.1365-2141.2003.04355.x 12780789

[4] Dumas G Merceron S Zafrani L Canet E Lemiale V Kouatchet A. Syndrome d’hyperviscosité plasmatique. La Rev Méd Interne (2015) 36:588–95. doi: 10.1016/j.revmed.2015.02.005 25778852

[5] Mehta J Singhal S. Hyperviscosity syndrome in plasma cell dyscrasias. Semin Thromb Hemost (2003) 29:467–71. doi: 10.1055/s-2003-44554 14631546

[6] Facon T Brouillard M Duhamel A Morel P Simon M Jouet JP. Prognostic factors in waldenström’s macroglobulinemia: a report of 167 cases. JCO (1993) 11:1553–8. doi: 10.1200/JCO.1993.11.8.1553 8336194

[7] PRUZANSKl W. Serum viscosity and hyperviscosity syndrome in IgG multiple myeloma: Report on 10 patients and a review of the literature. Ann Intern Med (1972) 77:853. doi: 10.7326/0003-4819-77-6-853 4644164

[8] Riccardi A Gobbi PG Ucci G Bertoloni D Luoni R Rutigliano L. Changing clinical presentation of multiple myeloma. Eur J Cancer Clin Oncol (1991) 27:1401–5. doi: 10.1016/0277-5379(91)90020-E 1835856

[9] Lemaire A Parquet N Galicier L Boutboul D Bertinchamp R Malphettes M. Plasma exchange in the intensive care unit: Technical aspects and complications. J Clin Apher (2017) 32:405–12. doi: 10.1002/jca.21529 28146331

[10] Padmanabhan A Connelly-Smith L Aqui N Balogun RA Klingel R Meyer E. Guidelines on the use of therapeutic apheresis in clinical practice – evidence-based approach from the writing committee of the American society for apheresis: The eighth special issue. J Clin Apher (2019) 34:171–354. doi: 10.1002/jca.21705 31180581

[11] Talamo G Farooq U Zangari M Liao J Dolloff NG Loughran TP. Beyond the CRAB symptoms: A study of presenting clinical manifestations of multiple myeloma. Clin Lymphoma Myeloma Leuk (2010) 10:464–8. doi: 10.3816/CLML.2010.n.080 21156463

[12] Moreau P Attal M Hulin C Arnulf B Belhadj K Benboubker L. Bortezomib, thalidomide, and dexamethasone with or without daratumumab before and after autologous stem-cell transplantation for newly diagnosed multiple myeloma (CASSIOPEIA): a randomised, open-label, phase 3 study. Lancet (2019) 394:29–38. doi: 10.1016/S0140-6736(19)31240-1 31171419

[13] Facon T Kumar SK Plesner T Orlowski RZ Moreau P Bahlis N. Daratumumab, lenalidomide, and dexamethasone versus lenalidomide and dexamethasone alone in newly diagnosed multiple myeloma (MAIA): overall survival results from a randomised, open-label, phase 3 trial. Lancet Oncol (2021) 22:1582–96. doi: 10.1016/S1470-2045(21)00466-6 34655533

[14] Kumar S Paiva B Anderson KC Durie B Landgren O Moreau P. International myeloma working group consensus criteria for response and minimal residual disease assessment in multiple myeloma. Lancet Oncol (2016) 17:e328–46. doi: 10.1016/S1470-2045(16)30206-6 27511158

[15] Avet-Loiseau H Attal M Moreau P Charbonnel C Garban F Hulin C. Genetic abnormalities and survival in multiple myeloma: the experience of the intergroupe francophone du myélome. Blood (2007) 109:3489–95. doi: 10.1182/blood-2006-08-040410 17209057

[16] Palumbo A Avet-Loiseau H Oliva S Lokhorst HM Goldschmidt H Rosinol L. Revised international staging system for multiple myeloma: A report from international myeloma working group. J Clin Oncol (2015) 33:2863–9. doi: 10.1200/JCO.2015.61.2267 PMC4846284 26240224

[17] Korn EL. Censoring distributions as a measure of follow-up in survival analysis. Stat Med (1986) 5:255–60. doi: 10.1002/sim.4780050306 3738291

[18] Preston FE Cooke KB Foster ME Winfiel DA Lee D. Myelomatosis and the hyperviscosity syndrome. Br J Haematol (1978) 38:517–30. doi: 10.1111/j.1365-2141.1978.tb01077.x 646950

[19] Armillas-Canseco F Hernández-Mata C Gómez-Ruiz I Aguayo A Martínez-Baños D. Hyperviscosity syndrome: A 30-year experience in a tertiary referral center in Mexico city. Blood (2015) 126:4741. doi: 10.1182/blood.V126.23.4741.4741

[20] Capra JD Kunkel HG. Aggregation of γG3 proteins: relevance to the hyperviscosity syndrome. J Clin Invest (1970) 49:610–21. doi: 10.1172/JCI106272 PMC322510 5415687

[21] Virella G Hobbs JR. Heavy chain typing in IgG monoclonal gamopathies with special reference to cases of serum hyperviscosity and cryoglobulinaemia. Clin Exp Immunol (1971) 8:973–80. PMC1713040 4997073

[22] Abeykoon JP Zanwar S Ansell SM Winters J Gertz MA King RL. Predictors of symptomatic hyperviscosity in waldenström macroglobulinemia. Am J Hematol (2018) 93:1384–93. doi: 10.1002/ajh.25254 30121949

[23] Blimark CH Turesson I Genell A Ahlberg L Björkstrand B Carlson K. Outcome and survival of myeloma patients diagnosed 2008–2015. real-world data on 4904 patients from the Swedish myeloma registry. Haematologica (2018) 103:506–13. doi: 10.3324/haematol.2017.178103 PMC5830385 29217784

