# Supplementary material for: Prognosis of hyperviscosity syndrome in newly diagnosed multiple myeloma in modern-era therapy: A real-life study
Source: Front Immunol. 2022 Dec 8;13:1069360. doi: 10.3389/fimmu.2022.1069360 (PMC9771682; doi:10.3389/fimmu.2022.1069360)

**Prognosis of hyperviscosity syndrome in newly diagnosed multiple myeloma in modern-era therapy: a real-life monocentric study**

Pierre-Edouard Debureaux MD^1^, Stéphanie Harel MD^1^, Nathalie Parquet MD^2^, Virginie Lemiale MD^3^, Virginie Siguret PhD^4^, Laurie Goubeau^4^, Florence Morin MD^5^, Bruno Royer MD^1^, Wendy Cuccuini MD, PhD^6^, Dikelele Elessa MD^1^, Floriane Theves MD^1^, Anne C Brignier MD Msc^2^, Elie Azoulay MD PhD^3,7^, Bertrand Arnulf MD, PhD^1,7^, Alexis Talbot MD^1,7^

Running title: Prognosis of hyperviscosity syndrome in myeloma

^1^Immuno-Hematology, Saint Louis Hospital, Paris, France

^2^Apheresis Center, Saint Louis Hospital, Paris, France

^3^Medical ICU unit, Saint Louis Hospital, Paris, France

^4^Hematology Laboratory, Lariboisière Hospital, Paris, France

^5^Immunology Laboratory, Saint Louis Hospital, Paris, France

^6^Cytogenetic laboratory, Saint Louis Hospital, Paris, France

^7^University Paris Cité, Paris, France

**Supplementary Table 1. Front-line multiple myeloma treatment for HVS NDMM patients**

|  | ≤65 years  N=25 | | >65 years  N=14 |
| --- | --- | --- | --- |
| Eligible to clinical trials for front line inclusion | | 16 (64%) | 9 (64%) |
| **Induction regimen type** | | | |
| VCD | 9 (36%) | | 5 (36%) |
| VTD | 6 (24%) | | 1 (7%) |
| VRD | 7 (28%) | | 4 (29%) |
| VMP | 0 | | 2 (14%) |
| Others | 3 (12%)* | | 1 (7%)** |
| Not treated | 0 | | 1 (7%) |
| Thrombosis during the front line | 2 (8%) | | 1 (7%) |
| AutoHSCT in the front line | 21 (84%) | | 1 (7%) |
| **Reason for not performing auto-HSCT** | 4 (1%) | | ND |
| No health care protection | 1 (4%) | | ND |
| Early progression | 2 (8%) | | ND |
| Trial design | 1 (4%) | | ND |
| AlloHSCT in the front line | 1 (4%) | | ND |
| **Best response to front-line therapy***** | | | |
| Complete response | 12 (46%) | | 1 (7%) |
| Very good partial response | 7 (28%) | | 5 (36%) |
| Partial response | 6 (24%) | | 4 (29%) |
| Stable disease | 0 | | 2 (14%) |
| None, refractory disease | 0 | | 1 (7%) |

AlloHSCT: allogeneic hematopoietic stem cell transplantation, AutoHSCT: autologous hematopoietic stem cell transplantation, VCD: bortezomib + cyclophosphamide + dexamethasone, VRD: bortezomib + lenalidomide + dexamethasone, VTD: bortezomib + thalidomide + dexamethasone, VMP: bortezomib + melphalan + dexamethasone

*one Daratumumab-VRD, one cyclophosphamide + thalidomide + dexamethasone and one sequence of bortezomib + driamycin + dexamethasone followed by VCD **one Daratumumab-VRD ***Response to first-line therapy was assessed by using the International Myeloma Working Group criteria^15^

|  | HVS (n=38): 18 deaths | Controls (n=76): 21 deaths |
| --- | --- | --- |
| Active multiple myeloma at death | **16 (89%)** | **19 (90%)** |
| Myeloma progression | 10 (56%) | 12 (56%) |
| Fatal bleeding unrelated to HVS | 2 (11%) | 1 (5%) |
| Infectious complication | 4 (22%) | 5 (24%) |
| SARS-CoV-2 infection | 0 | 1 (5%) |
| Death at multiple myeloma remission | **2 (11%)** | **2 (10%)** |
| Transplant related mortality | 2 (11%)* | 0 |
| Chronic respiratory failure | 0 | 1 (5%) |
| Unrelated cancer | 0 | 1 (5%) |

**Supplementary Table 2. Cause of death in HVS and control multiple myeloma cohorts**

**HVS: hyperviscosity syndrome**

*Interstitial pneumopathy post 2^nd^ autologous stem cell transplantation (n=1) and multi-organ failure post allogeneic stem cell transplantation (n=1)

**Supplementary Table 3. Univariate analysis for OS in NDMM with HVS at diagnosis**

| Variable | HR (95%CI, p-value) |
| --- | --- |
| Women (vs Men) | 1.83 (0.69–4.83, p=0.22) |
| Age >65 years (vs ≤65 years) | 0.73 (0.24–2.25, p=0.59) |
| ECOG > 1 (vs ECOG 0–1) | 2.65 (0.80–8.79, p=0.11) |
| M-protein level at diagnosis | 0.98 (0.95–1.01, p=0.16) |
| IgG (vs IgA) | 0.64 (0.23–1.75, p=0.38) |
| % of plasma cells in bone marrow | 1.00 (0.98–1.01, p=0.71) |
| **>5% of plasma cells in blood** | **6.06 (2.10–17.50, p=0.01)** |
| **High-risk cytogenetics** | **2.98 (1.14–7.80, p=0.03)** |
| t(4;14) | 2.13 (0.74–6.08, p=0.16) |
| Del (17p) | 2.01 (0.45–8.87, p=0.36) |
| t(11;14) | 0.54 (0.19–1.56, p=0.26) |
| 1q + | 1.95 (0.63–6.03, p=0.24) |
| Del (1p32) | 1.27 (0.16­–10.05, p=0.83) |
| **LDH above the normal level** | **3.66 (1.32–10.18, p=0.01)** |
| ISS3 (versus ISS1-2) | 1.90 (0.65–5.53, p=0.24) |
| **R-ISS3 (versus R-ISS1-2)** | **3.33 (1.07–10.34, p=0.04)** |
| **Initial hospitalization in ICU** | **7.24 (2.29–22.9, p=0.001)** |
| AutoHCST realization | 1.03 (0.38–2.79, p=0.95) |

ECOG: Eastern Cooperative Oncology Group, M-protein: monoclonal immunoglobulin, ISS: International staging system, RISS: revised ISS, ICU: intensive care unit, AutoHSCT: autologous hematopoietic stem cell transplantation

**Supplementary figure 1. Chronological evolution of HVS patients based on therapeutic choice**

1. Evolution of protein level for the three patients who have not received TPE.
2. Evolution of protein level for patients who received TPE (before TPE, after one TPE, and after two TPE). Two patients with star symbol in ICU increased protein levels after the second TPE related to high compensation volume of fresh plasma. No difference for protein level decrease was observed between IgG and IgA myeloma (-28% vs. -25%, p=0.20). No difference for protein level decrease was observed between albumin and FFP or albumin alone for fluid compensation (median ­­­-24% vs. -21%, p=0.61). Median protein levels decreased by 24% and 32% after one and two TPE sessions, respectively.
3. Evolution of protein level for patients who received TPE (before TPE, one day after last TPE and one month after TPE). One patient with star symbol had increase at 1 month after TPE related to refractory MM.


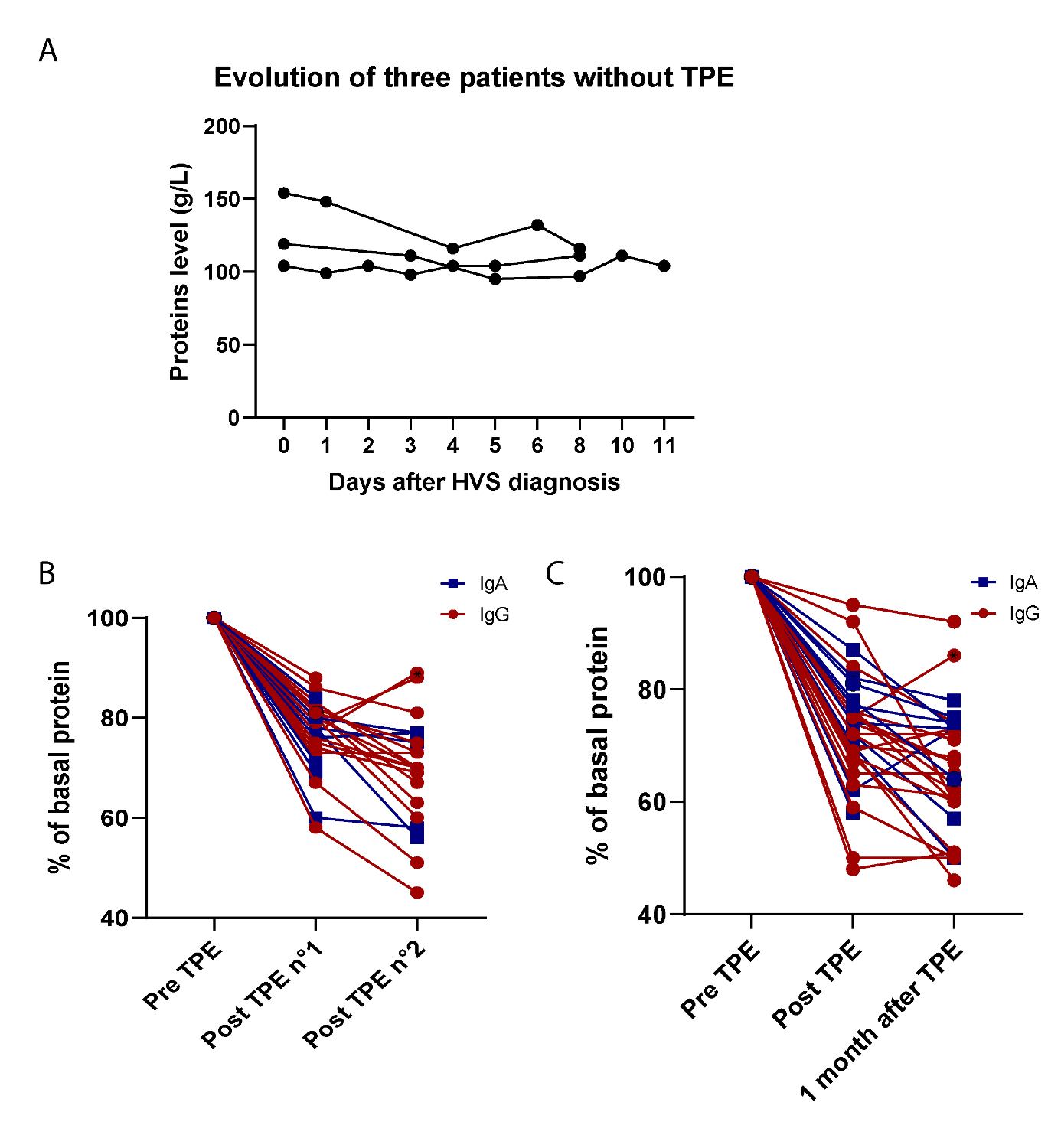

Supplement: Supplementary file 1 [file DataSheet_1.docx]
